# Flood Impacts in Keppel Bay, Southern Great Barrier Reef in the Aftermath of Cyclonic Rainfall

**DOI:** 10.1371/journal.pone.0084739

**Published:** 2014-01-10

**Authors:** Alison M. Jones, Ray Berkelmans

**Affiliations:** 1 Central Queensland University, Rockhampton, Queensland, Australia; 2 Australian Institute of Marine Science, Townsville Mail Centre, Queensland, Australia; National University of Singapore, United States of America

## Abstract

In December 2010, the highest recorded Queensland rainfall associated with Tropical Cyclone ‘Tasha’ caused flooding of the Fitzroy River in Queensland, Australia. A massive flood plume inundated coral reefs lying 12 km offshore of the Central Queensland coast near Yeppoon and caused 40–100% mortality to coral fringing many of the islands of Keppel Bay down to a depth of ∼8 m. The severity of coral mortality was influenced by the level of exposure to low salinity seawater as a result of the reef's distance from the flood plume and to a lesser extent, water depth and whether or not the reef faced the plume source. There was no evidence in this study of mortality resulting from pollutants derived from the nearby Fitzroy Catchment, at least in the short term, suggesting that during a major flood, the impact of low salinity on corals outweighs that of pollutants. Recovery of the reefs in Keppel Bay from the 2010/2011 Fitzroy River flood is likely to take 10–15 years based on historical recovery periods from a similar event in 1991; potentially impacting visitor numbers for tourism and recreational usage. In the meantime, activities like snorkeling, diving and coral viewing will be focused on the few shallow reefs that survived the flood, placing even further pressure on their recovery. Reef regeneration, restoration and rehabilitation are measures that may be needed to support tourism in the short term. However, predictions of a warming climate, lower rainfall and higher intensity summer rain events in the Central and Coastal regions of Australia over the next decade, combined with the current anthropogenic influences on water quality, are likely to slow regeneration with consequent impact on long-term reef resilience.

## Introduction

Cyclonic rain events are an intrinsic factor shaping the nature, location and extent of inshore coral reefs of the Great Barrier Reef (GBR). A large part of the damage to inshore coral communities from cyclones is caused by floodwater inundation of reefs adjacent to the major catchments with low salinity exposure influenced largely by the dynamic movement of the flood plume as a result of wind-derived currents and tides [1]. Large loads of suspended sediment and particulate nutrients are also discharged in high flow events from erosion in grazing lands and dissolved inorganic nutrients from fertilised cropping lands in adjacent catchments [2]. It seems logical that the pattern of coral mortality caused by these events correlates strongly with the extent of a reef's exposure to freshwater [3] in the short term and exposure to pollutants in the longer term. In fact, it can be said that the spatial patterns of coastal and inshore reef development on the GBR is largely determined by their distance from the source of runoff and floodwaters. Because of the inherent difficulties in monitoring reefs during conditions of high turbidity caused by sediment re-suspension, few studies have investigated the spatial pattern and extent of their short term impact. Natural disturbance events such as floods are not currently incorporated into reef management decisions but given their potential for shaping the nature of inshore reefs, understanding the patterns and severity of flood impacts can inform predictions of recovery that then help assess the potential effects on industries such as local tourism as well as conservation efforts.

**Figure 1:**
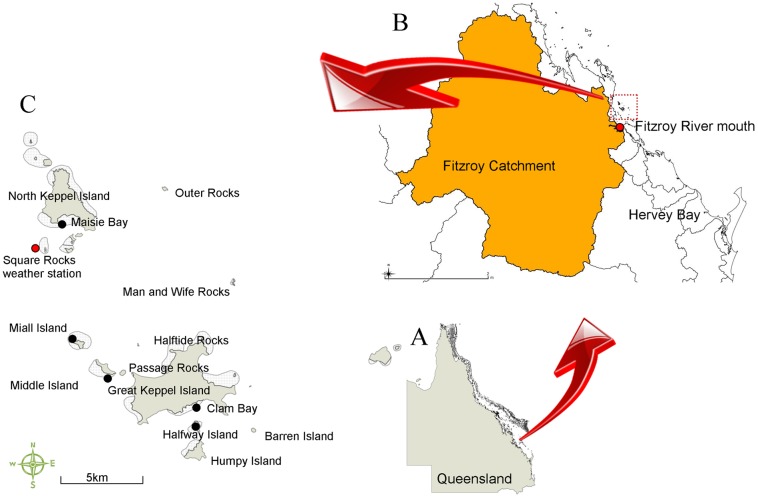
Map of the study site. Map of Queensland (Inset A) showing the size of the Fitzroy Catchment (orange polygon, Inset B) and the mouth of the Fitzroy River (red dot, Inset B) in relation to the location of the five monitoring sites in Keppel Bay (Inset C) and the Australian Institute of Marine Science Square Rocks weather station (red dot Inset C).

**Figure 2:**
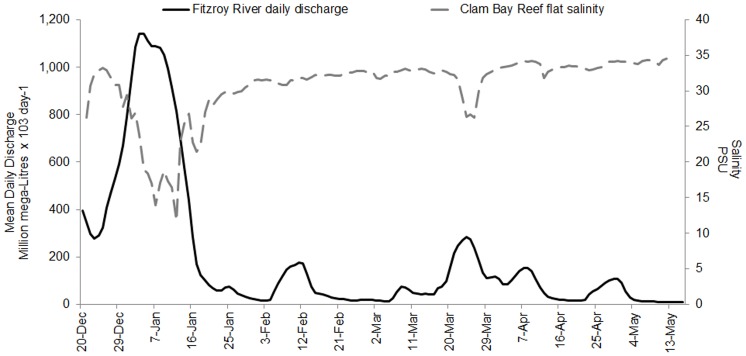
Time-series of Fitzroy River discharge and Clam Bay salinities. Time-series of mean daily discharge in million ML × 10^3^ day^−1^ (solid line) of the Fitzroy River (dotted line), and salinity in PSI measured on Clam Bay reef flat (dashed line, 0–2.0 m at chart datum, 2.4–4.4 m at mean sea level), showing the peak of the flood between 01 January and 15 January 2011 which preceded the low salinity event in Keppel Bay by ∼9 days (Fitzroy River discharge data was sourced from the Queensland Government at http://watermonitoring.derm.qld.gov.au). The shaded area illustrates the approximately 9 day lag between peak discharge and the measured lowest salinity at Clam Bay.

The summer of 2010-11 brought with it heavy rain from Cyclones ‘Tasha’ and ‘Yasi’ that resulted in flooding of several major catchments along the Queensland coast. Tropical Cyclone ‘Tasha’ crossed the coast near Babinda in North Queensland on 24 December 2010. The worst coral mortality occurred in the southern GBR (Keppel Bay Islands) and ∼300 km south in the Sandy Straits Marine Park between Fraser Island and Hervey Bay [4] whereas most other inshore GBR reefs escaped severe impacts [5]. In the Central Queensland region, between December 2010 and February 2011 the Fitzroy River reached a peak mean daily discharge of 1.16 million mega-litres day^−1^ [6] over a period of ∼18 days, resulting in a large flood plume entering the adjacent Keppel Bay (Fig 1,2) and causing extensive coral mortality on reefs fringing the islands [7], [8]. Agricultural herbicides such as tebuthiuron, atrazine and diuron that are capable of inhibiting photosynthesis in marine benthic organisms were found in discharges from the Fitzroy River in 2011 [8]; mirroring a previous high flow event in 2008 [9], [10]. A similar flood event occurred in the Fitzroy in 1991 as a result of Cyclone ‘Joy’. The peak mean daily discharge for the 1991 event was 1.14 million ML day^−1^ with the event lasting ∼13 days. The 1991 flood resulted in 30–90% coral mortality to depths of 0.5–2.3 m below chart datum (2.9–4.7 m below mean sea level for Keppel Bay) in a pattern consistent with distance from, and exposure to, the plume [9], [11], [12]. By 2008, the reefs had recovered to ∼52% overall hard coral cover, 17 years after the flood [13]. Such major floods can have devastating impacts on the regional economy because of the loss to tourism and infrastructure [14]. While tourist visitation does not depend entirely on the existence of healthy coral reefs, recovery to their pre-flood state, or better, is critical in supporting reef-based tourism, recreation and the resilience of the reef system as a whole.

The Keppel Islands in the southern GBR support a significant marine tourism industry, with most snorkelling, diving, swimming, fishing and coral-viewing focused near the expansive (up to 200 m wide) shallow platform reefs. A number of commercial tourist vessels service the Bay's 17 islands from Rosslyn Bay harbour on the adjacent mainland which also serves as the main departure point for recreational vessels. These wide, shallow platform reef flats have developed on the protected southern and western sides of most of the islands with an average depth of ∼1.3 m below chart datum [12] and, until the flood, were composed predominantly of branching acroporids and pocilloporids [13]. The reef flats gradually merge into deeper coral thickets dominated by branching *Acropora* that rise from a sandy substrate, providing ideal habitat for recreationally and commercially valuable reef fish like coral trout [15].

The proximity of the Keppel Islands to the mainland coast (∼12 km) makes Keppel Bay fringing reefs highly accessible. Their accessibility has encouraged the development of industries and activities such as fishing, tourism and local recreation whose short- and long-success depends on their resilience. Population growth in the region has increased by an average 1.6% per annum [16], [17] with a concurrent 19% rise in recreational vessel registrations since 2009 and a 60% increase in commercial tourist visitation over the last decade [18], [19]. While healthy coral reefs are not the only attractant in the Bay, a recent survey of visitors to the GBR rated encounters with coral and marine life as highly important; with 34% nominating snorkelling, and 13% nominating diving as their best visitor experiences [20], [21]. Clearly, healthy coral reefs are of great economic and social value to the region. The commercial and recreation value of the Keppel Islands reefs, along with predictions of a warmer climate have raised management concerns about the cumulative impacts of flooding, bleaching [22] and water quality on the continued resilience of the region's reefs. It is now well understood that as well as floods causing coral mortality the interaction between low salinity water and the composition of the water in terms of turbidity, suspended sediments (and sedimentation), nutrients and phytoplankton, and pesticides are critical to coral stress, bleaching response, and mortality [23]–[26]. Managers may need to consider the spatial impacts of major floods and their interaction with major tourism infrastructure in conservation efforts within the next decade.

In this study, we report the impacts of a record rainfall event associated with Cyclone ‘Tasha’ on southern GBR inshore reefs in Keppel Bay and investigate some of the spatial, temporal and environmental factors potentially contributing to the severity of these impacts. We discuss the implications of these factors for recovery trajectories, conservation planning for resilience and marine tourism.

## Methods

### 1. Study sites

The Keppel Bay Islands lie ∼30 km from the mouth of the Fitzroy River near Rockhampton and Yeppoon in Central Queensland. The Fitzroy River drains the largest catchment of the GBR coast in the south of the Bay [27]. Reefs fringing islands to the north of the Fitzroy River, in Keppel Bay, have previously been described in detail in Jones and Berkelmans [13].

To investigate the spatial, temporal and environmental impacts of the 2011 Fitzroy River flood caused by Cyclone ‘Tasha’ on reefs in Keppel Bay, five reef areas that were likely to be impacted by the flood and were lying in a continuum of distance from the mouth of the Fitzroy River were chosen as monitoring sites. Because upwelling behind islands has been shown to partially protect reefs from fresh polluted water [28], [29], four of the reefs chosen faced the direction of the plume source (southern and western sides of islands including North Keppel Island, Middle Island, Great Keppel Island at Clam Bay and Halfway Island, Table 1), and one reef was protected from the full impact of the plume by Miall Island. At the time of the study design, these sites were expected to follow a similar pattern of impact to that of the flood in 1991 which dissipated as it was driven north by moderate south easterly winds.

**Table 1:** Details of the 5 sites in the flood impact study showing reef flats (0–2.0 m at chart datum, 2.4–4.8 m at mean sea level) and reef slopes (6.0–12.0 m at chart datum, 8.4–14.4 m at mean sea level), latitudes and longitudes, distance (‘near’  =  ∼40 km, ‘middle’  =  ∼43 km and ‘far’  =  ∼50 km) from the source of the flood plume at the mouth of the Fitzroy River ), and reef aspect (North, West or South).

Site	Depth	Latitude	Longitude	Reef Aspect	Distance
Great Keppel Island (Clam Bay)	flat	−23.1859	150.9749	South	Near
	slope	−23.1859	150.9749	South	Near
Halfway Island	flat	−23.199	150.9700	West	Near
	slope	−23.1989	150.9697	West	Near
Miall Island	flat	−23.1498	150.9036	North	Middle
	slope	−23.1498	150.9036	North	Middle
Middle Island	flat	−23.1713	150.9217	South	Middle
	slope	−23.1713	150.9217	South	Middle
Nth Keppel Island	flat	−23.0858	150.8964	South	Far
	slope	−23.0858	150.8964	South	Far

### 2. Salinity

Following predictions of major flooding of the Fitzroy River, on 20 December 2010, conductivity/temperature loggers (Odyssey Dataflow systems, New Zealand) were deployed at two depths at each of the five sites: reef flats (0–2.0 m at chart datum, 2.4–4.4 m at mean sea level) and reef slopes (6.0–12.0 m at chart datum, 8.4–14.4 m at mean sea level). Time-series of mean daily water discharge at the Gap Gauging Station along the Fitzroy River was determined from data sourced from the Queensland Government (http://watermonitoring.derm.qld.gov.au). This station is ∼100 km upstream from the mouth of the Fitzroy River. Conductivity (mS cm^−1^) measurements loggers deployed at the study sites were converted to practical salinity units (PSU) using the using the UNESCO-adopted algorithm of Lewis [30]. Technically, the converted salinity values are dimensionless but for the sake of clarity they are hereafter followed by the “PSU” abbreviation.

### 3. Coral condition

At each of the five study sites, changes in coral condition and cover were recorded along three 20 m long × 1 m wide transects at on reef flats and slopes at the same depths and locations as the salinity loggers. The percentage of living coral cover was recorded before the flood on 20–21 December 2010 and again, after salinities had returned to normal on 15 May 2011. In between these surveys, on 2 January, 21 February, 3–4 March when the flood plume had enveloped the Keppels, general notes on the condition of the coral communities were recorded as visibility allowed. At the time of the last survey most corals had either recovered from bleaching or died. Mortality data only included colonies where death was assumed to be recent and flood-related. Recent death was attributed based on changes from previous surveys and the presence of coral skeletons recently colonised by macro algae.

For the mortality surveys, at each time point, site and depth, three replicate transects were photographed using a 4 mega-pixel digital camera fitted with a 16 mm wide-angle lens at 1 m intervals. Geo-referenced images were obtained for each transect with a towed GPS using the methods of Roelfsema and Phinn [31] which enabled repeat surveys to be conducted within a few metres of their original location. Digital still images were analysed using 10 random points per image with the program CPCe^TM^ v3.1 [32].

### 4. Flood imagery and weather

To assess the impact of the flood against environmental conditions, Moderate Resolution Imaging Spectroradiometer (MODIS) Aqua true colour satellite images of the Fitzroy flood plume were downloaded from the NASA website at (250 m resolution -http://lance-modis.eosdis.nasa.gov/imagery/subsets/?project=other&subset=Australia3). Wind conditions, tides and water temperatures for the 2011 flood were sourced from the Australian Institute of Marine Science Square Rocks weather station [33].

### 5. Photosystem II inhibiting herbicide concentrations

PSII herbicides are plastoquinone analogs that inhibit photosynthesis by reversibly binding to the Quinone-B binding site on the D1 protein [34]. To assess the impact of exposure to 12 PSII herbicides on coral communities at four of the five sites, passive samplers were deployed between 2 January and the 8 February 2011 (excluding Miall Island). Full details of the entire suite of pesticides samples can be found in Kennedy et al. [8]. Only herbicides found in significant concentrations and with the potential for PSII inhibition are included in this study because of their relevance to scleractinian coral-symbiont photosynthesis [23] and reproduction [35], [36]. Time-integrated concentrations (ng.L^−1^) for 9 of the herbicides were converted to PSII-HEq (equivalent to diuron concentrations derived using average relative potencies) for each site (Table 2) as outlined in Kennedy et al. [8], [37], [38]. Maximum PSII-HEq is an index widely accepted on the GBR for potential for photosynthetic inhibition of diatoms, seagrass and coral-symbionts with respect to the effects of diuron [39].

**Table 2:** List of time-averaged concentrations (ng.L^−1^) of herbicides found over one month's deployment at four sites in Keppel Bay and their photosystem II inhibition potential based on relative potency (potency factor*) with respect to the reference diuron.

Pollutant name	Potency factor*	Middle Is	North Keppel Is	Halfway Is	Gt Keppel Is (Clam Bay)
Ametryn	1.31		0.27		
Atrazine	0.16	5.91	7.96	7.94	5.37
Atrazine desethyl	0.11	0.91	1.18	1.47	0.69
Diuron	0.003	5.03	7.92	5.81	4.14
Hexazinone	1	1.31	1.75	1.98	1.04
Metolachlor	0.38	2.52	3.18	3.46	2.06
Prometryn	1.05	0.29	0.42	0.36	0.13
Simazine	0.07	0.63	0.74	0.92	0.58
Tebuthiuron	0.08	16.61	20.27	23.33	13.24
**PSII-HEq** **		8.3	12	10.3	6.7

Photosystem II equivalent concentrations (PSII-HEq) were calculated for each site by summing the PSII-HEq for individual herbicides**.

Relative potency factors of each of the herbicide with respect to the reference diuron, in terms of PSII inhibition.

PSII-HEq were calculated by summing individual PSII-HEq for each herbicide derived by multiplying the time-averaged concentration in water by the relative potency with respect to the reference herbicide diuron [37].

### 6. Statistical analysis

To investigate the spatial, temporal and environmental variables influencing coral mortality, a Euclidian distance matrix of the similarities in the percentage of coral mortality (the difference between the percentage of live coral cover in December 2010 and that in May 2011) between the five sites was subjected to a multivariate regression procedure (DISTLM, [40]) using PERMANOVA v1.03 [41]–[43].

To determine the most appropriate regression model, 999 permutations of the spatial (distance, depth and reef aspect), temporal (the number of days corals were exposed to salinities of 22–28 PSU) and environmental (PSII-HEq max) variables were tested to investigate the best combinations that explained differences in the percentage of coral mortality at the sites. The results from the model were displayed in a 2-D, distance-based redundancy bi-plot. For the factor ‘distance’, sites were categorised as ‘near’ (∼40 km), ‘middle’ (∼43 km) and ‘far’ (∼50 km) from the source of the flood plume at the mouth of the Fitzroy River. For the factor ‘reef aspect’, sites were categorised as south, west (facing the flood plume source) or north (behind the island), and for ‘depth’ sites were categorised as either reef flat or reef slope. Middle Island salinity data had a gap for a few weeks during the peak of the flood and for the purpose of the analysis, salinities during this period were assumed to be similar to neighbouring Miall Island (1.5 km west of Middle Island) based on distance from the flood plume (Fig 1). Similarly, passive samplers were not deployed at Miall Island and for the purpose of the analysis, PSII-HEq was assumed to be the same as those at Middle Island. PSII-HEq was also assumed to be independent of depth.

This study was made possible by funding provided by Central Queensland University and permitted by the Great Barrier Reef Marine Park Authority.

## Results

From 01 December 2010 to 01 May 2011, the highest recorded Queensland rainfall [44] associated with Tropical Cyclone ‘Tasha’ caused the Fitzroy River to discharge an estimated 35.3 km^3^ of fresh water into Keppel Bay with peak flow between 01 and 15 January 2011 (Fig 2). The peak discharge of the 2011 flood event was ∼13,000 cubic metres sec^−1^ (cumecs) compared with ∼15,000 cumecs for the 1991 event.

**Figure 3:**
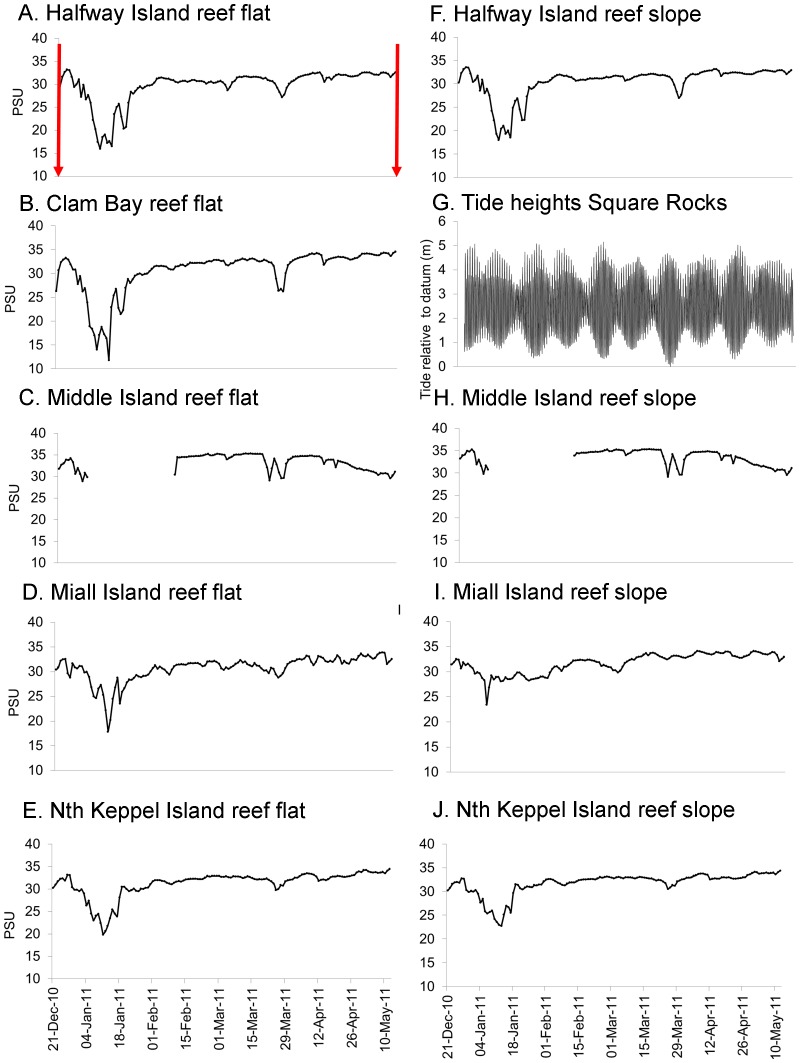
Real-time salinities at the study sites in Keppel Bay. Average daily real-time salinities measurements (PSU  =  practical salinity units) on reef flats at A. Halfway Island, B. Clam Bay, C. Middle Island, D. Miall Island and E. North Keppel Island and on reef slopes at F. Halfway Island, H. Middle Island, I. Miall Island and J. North Keppel Island (data for Clam Bay slope were not available) and G. tide heights (Lowest Astronomical Tide, chart datum) at Square Rocks weather station 21 December 2010 to 10 May 2011. The sites are shown in a continuum of increasing distance from the Fitzroy River mouth. The two time points at which coral condition was recorded for this study (December 2010 and May 2011) are marked with a red arrow.

Salinity levels at the study sites before the flood were between 33–35 PSU (Fig 3) Keppel Bay reef flat and slope salinity levels lagged Fitzroy River average daily discharge levels by ∼9 days with average daily salinities below 30 PSU at Halfway Island (6 m, reef slope) occurring for ∼3 weeks between 1 January and 23 January 2011. The lowest salinity was recorded on 12 January 2011 on the reef flat (2 m) at Clam Bay with half-hourly values as low as 6.5 PSU and an average daily salinity of 11.8 PSU. This site experienced 5 days of salinities below 30 PSU.

**Figure 4:**
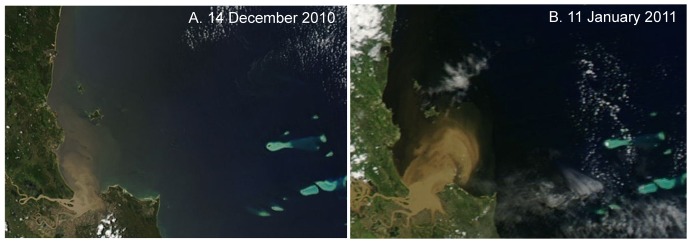
Pseudo true-colour images of the Fitzroy River flood plume. Pseudo true-colour images of the progress of the flood plume of the Fitzroy River on A. 14 December 2010 and B. the 11 January 2011 captured by Moderate Resolution Imaging Spectroradiometer (MODIS) Aqua satellite.

**Figure 5:**
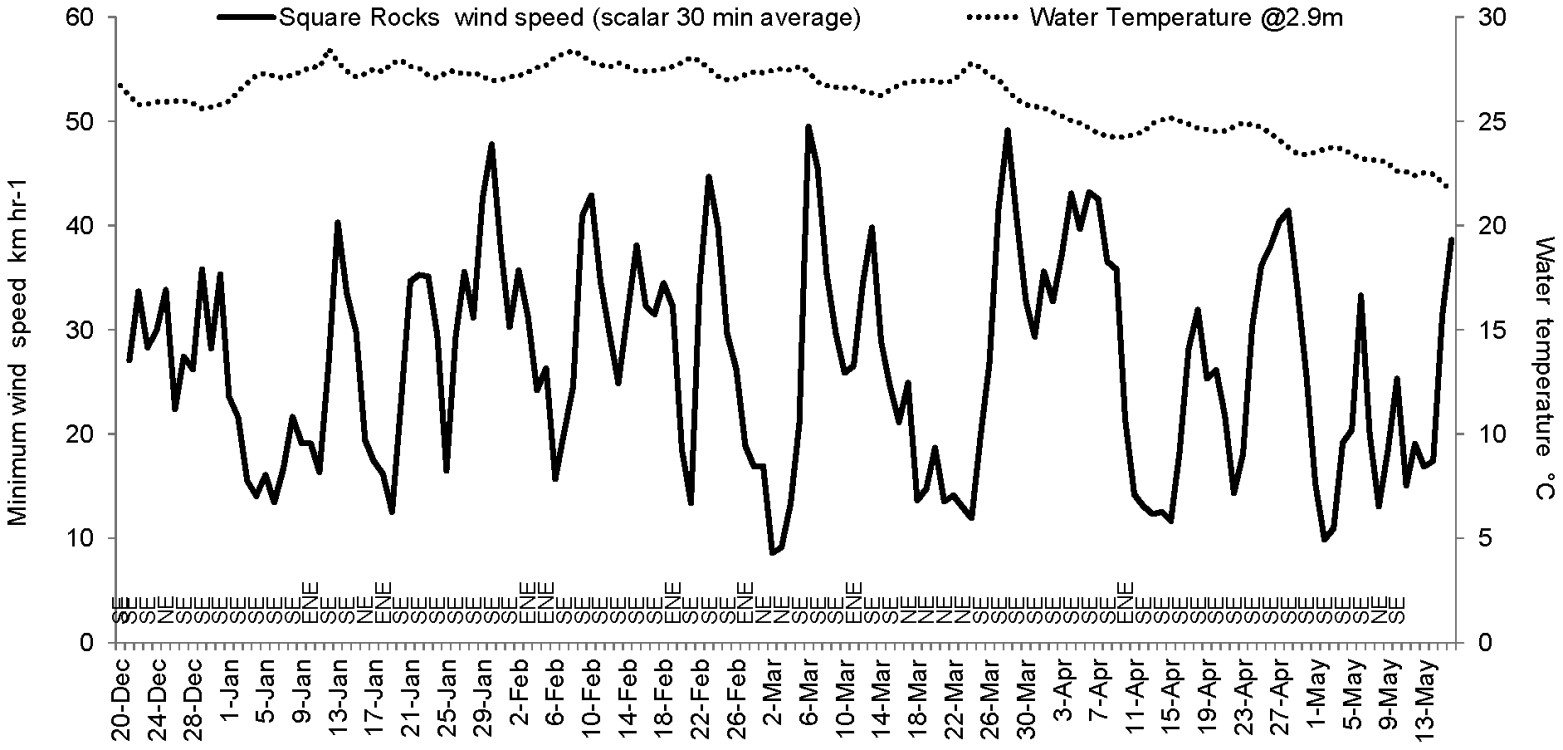
Minimum wind speed and water temperature in Keppel Bay during the study. Time-series of minimum wind speed in km hr^−1^ (solid line) and water temperature in °C (dotted line) at Square Rocks in Keppel Bay showing the prevailing south-easterly wind direction during the Fitzroy River flood between 20 December 2010 and 14 May 2011 [33].

Floodwaters from the Fitzroy River dispersed into Keppel Bay in a ∼200 km^2^ plume stretching 70 km northwards that was visible in imagery captured by MODIS Aqua on 14 December 2010 and 11 January 2011 (Fig 4a, b). Between 3 and 7 January, when reefs experienced lowest salinity, wind conditions in Keppel Bay were <15 knots, rising to 28 knots on 13 January from the south east (Fig 5). Light northerly winds again prevailed on 17 January for a few days, rising to 25 knots from the south east on 21 January. Between 3 and 7 January, Spring tides prevailed with a mean tidal range of ±3.9 m and low tide levels between 0.6 m and 0.9 m above chart datum. Neap tides prevailed from 10 to 13 January with a mean tidal range of ±1.6 m and low tide level of 1.6 m (Fig 3j). Water temperatures varied between 26°C and 27°C during the flood event (26 December 2010 to 18 January 2011, Fig 5).

Before the flood, coral cover at Middle Island, Miall Island, Halfway Island and Great Keppel Island (Clam Bay) was comprised predominantly of *Acropora*, *Pocillopora*, fungiids, *Turbinaria* and soft corals ranging from 17–70% cover on reef flats and 20–56% on reef slopes. By 21 December 2010, Acropora corals at Halfway Island, the closest site to the Fitzroy River mouth and facing the plume source, were pale in colouration and some colonies showed signs of tissue sloughing and mucous discharge. Corals at sites further from the plume source were unaffected at this time. On 8 February 2011, almost all corals at North Keppel Island were bleached white and corals at Clam Bay (which were unaffected on 21 December 2010) were recently dead and covered with a fine layer of turf algae. At Miall Island, facing away from the plume source, corals were bleached white when observed on 21 February 2011 but many had died by 3 March 2011.

**Figure 6:**
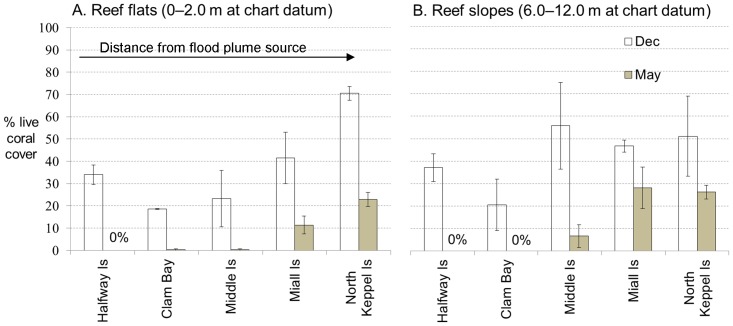
Percentage coral cover on reef flats and slopes in Keppel Bay during the study. Percentage coral cover on A. reef flats and B. reef slopes at the five study sites in the Keppels before (December 2010) and after (May 2011) a major flood in the Fitzroy Catchment as a result of Cyclone ‘Tasha’ shown in a continuum of increasing distance from the flood plume source. The bars represent the mean % coral cover of three replicate transects on reef flats and reef slopes as a percentage of the total benthic cover before the flood in December 2010 (grey bars) and then in May 2011 (white bars), after the flood peak. Error bars represent the standard errors of the means.

By May 2011, after the flood plume had dissipated, fringing reefs in the inner, southern section of Keppel Bay, closest to the river mouth (Halfway Is and Clam Bay) had suffered 40–100% coral mortality on reef slopes and 68–100% on reef flats (Fig 6). Reefs fringing outer islands of the Bay (Barren Island and Outer, Man and Wife, and Egg Rocks) were unaffected by the flood. Miall Island lost the least amount of living coral (40–68% for reef slope and flat respectively) while Great Keppel Island (Clam Bay) and Halfway Island lost 100% of their pre-flood coral cover to ∼8 m depth. North Keppel Island lost 49% and 68% and Middle Island lost 88% and 99% of pre-flood live coral cover on reef flats slopes respectively. Reefs at Pelican Island, which is closer to the mouth of the river than the five study sites, were also severely impacted, with almost 100% mortality to approximately ∼6 m depth and variable mortality below this depth (A. Thompson, personal communication).

A range of pesticides or pesticide transformation products were detected in passive samplers deployed during the Fitzroy flood event at four of the five study sites (Table 3). A full description of these can be found in Kennedy et al. [8]. Surprisingly, PSII-HEq appeared to increase with increasing distance from the flood plume source, ranging from 6.7 ng.L^−1^ at Great Keppel Island (Clam Bay) to 12.5 ng.L^−1^ at North Keppel Island (Table 2). PSII-HEq index at Middle Island and Clam Bay fell within category 5 (PSII-HEq ≤ 10 ng.L^−1^) whereas North Keppel Island and Halfway Islands PSII-HEq index fell within category 4 (10 < PSII-HEq ≤ 50 ng.L^−1^). Herbicide levels within PSII-HEq Catetory 5 are below that shown in any scientific studies that demonstrate effects on plants or animals based on toxicity or a reduction in photosynthesis. Herbicide concentrations within Category 4 are reported as having no scientific observations of reduced photosynthesis for two diatoms [37]. There are published studies of reduced photosynthesis for zooxanthellae (both isolated and *in hospite*) from three coral species 250 < PSII-HEq 900 ≤ ng.L^−1^ [37].

**Table 3:** Average concentrations of 9 herbicides detected in passive samplers deployed in duplicate at four sites in Keppel Bay between 2 January and 8 February 2011.

Site	Middle Is		North Keppel Is		Halfway Is		Gt Keppel Is (Clam Bay)	
	Avg	Std	Avg	Std	Avg	Std	Avg	Std
	ng.L^−1^		ng.L^−1^		ng.L^−1^		ng.L^−1^	
Ametryn			0.27	0				
Atrazine	5.91	0.89	7.96	0.87	7.94	0.86	5.37	1.13
Atrazine desethyl	0.91	0.00	1.18	0.00	1.47	0.00	0.69	0.19
Diuron	5.03	0.34	7.92	0.66	5.81	0.00	4.14	0.92
Hexazinone	1.31	0.00	1.75	0.23	1.98	0.00	1.04	0.21
Metolachlor	2.52	0.00	3.18	0.35	3.46	0.00	2.06	0.32
Prometryn	0.29	0.00	0.42	0.20	0.36	0.00	0.13	0.18
Simazine	0.63	0.18	0.74	0.00	0.92	0.00	0.58	0.16
Tebuthiuron	16.61	0.00	20.27	3.41	23.33	1.50	13.24	3.00

**Figure 7:**
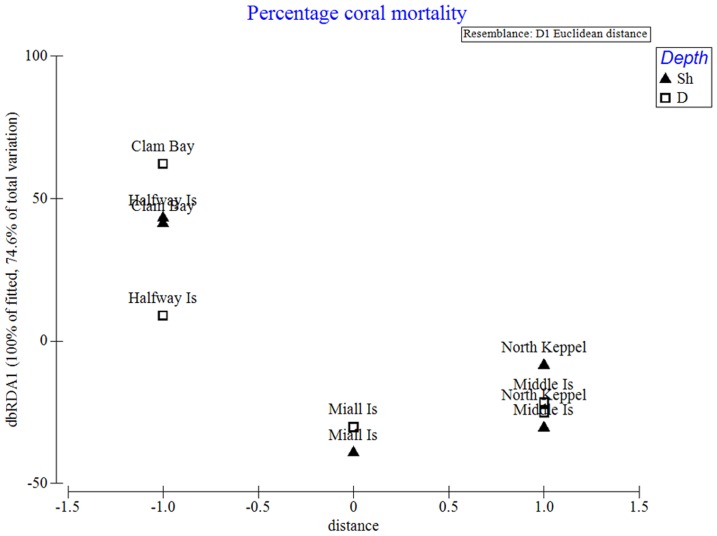
Redundancy-based distance analysis bi-plot of the study sites in Keppel Bay. A redundancy -based distance analysis bi-plot of flood-damaged sites in the Keppel Islands based on multivariate regression analysis of the best combinations of the explanatory variables (distance from the flood plume source, reef aspect, depth, the number of day's exposure to salinities from 22–28 PSU and PSII-HEq max) showing the significant influence of distance (shown along the x-axis) explaining 45% of the variation in coral mortality between sites. The redundancy plot was derived using the DISTLM procedure of Primer. Site names are shown beside each symbol (Clam  =  Clam Bay, NK  =  North Keppel Island, MID  =  Middle Island, MIALL  =  Miall Island, HW  =  Halfway Island).

Multiple regression analysis showed that the best explanatory variable for coral mortality was the distance of a site from the flood plume source (P<0.05) which explained 45% of the variability of the fitted model. The period of exposure to low salinity explained ∼20% of the variation (ns) with PSII-HEq max explaining ∼6% (ns) and reef aspect (south, north or west) and depth/habitat (reef flat or slope) explaining <1% of the variation (Table 4). Although not statistically significant, the influence of depth and low salinity could be seen in the (Euclidian) distance-based redundancy bi-plot of the study sites (Fig 7).

**Table 4:** Marginal tests of the best combination of explanatory variables following multiple regression analysis of five sites impacted by flooding in the Keppel Islands.

Variable	SS (trace)	Pseudo-F	P	% variance explained
distance	7271.8	6.5	0.049	45*
#days at 22 PSU	3303.3	2.1	0.207	20
#days23	3012.2	1.8	0.217	19
#days24	2334.4	1.3	0.288	14
#days25	1415.2	0.8	0.410	9
PSII-HEq max	1027.0	0.5	0.477	6
#days26	906.0	0.5	0.506	6
#days28	748.5	0.4	0.542	5
#days27	693.0	0.4	0.555	4
aspect	129.6	0.1	0.805	1
depth	13.7	0.0	0.992	0

Distance from the source of the flood plume explained 45% of the variation (P<0.05, marked with an asterisk *).

## Discussion

The 2011 Fitzroy flood caused by heavy rains from Tropical Cyclone ‘Tasha’ resulted in almost 100% loss of coral cover to at least 2 m depth and significant loss of living coral below 2 m and to a depth of ∼8 m on reefs on the southern and western sides of most inner islands of Keppel Bay. Coral mortality was high as a result of osmotic stress, and potentially additional stressors such as sediment and nutrients, but varied with reef aspect and distance from the river mouth. Reefs in the outer section of the Bay, furthest from the Fitzroy River mouth, and/or that were protected from the full effect of the floodwaters by the ‘island wake’ effect [28], [29] experienced the least coral mortality. The wide-spread and prolonged nature of the flood (∼18 days) meant salinity levels were beyond the predicted tolerance range of 22 PSU to 28 PSU for 3–16 days respectively for branching *Acropora* species [7]. Short-term mortality of corals was a result of exposure to hyposaline seawater which resulted in bleaching followed by tissue sloughing within hours or days [12]. PSII-HEq max values did not enter the range (250–900 ng.L^−1^) known to affect corals. The rapid rate of bleaching and mortality caused by decreased salinity appeared to outweigh any effects from PSII inhibiting pollutants.

The 2011 Fitzroy flood volume was less than that in 1991 [11], [12], however the impact to fringing reefs was greater because of the prevailing wind conditions. For instance, at Clam Bay and Halfway Island, 30–50% coral cover was left after the 1991 flood event [11] whereas after the 2011 flood almost no living coral cover was left at these sites even at a depth of 8 m. In Keppel Bay, south easterly winds produce a northward-moving current drift. As a result, moderate south-easterly winds coinciding with the flooding are likely to keep flood plumes close to the coast and travelling northwards away from the reefs. In 1991, these wind conditions probably reduced the exposure of corals to low salinities at some sites until well after the peak discharge when the plume began moving to the east [45]. During the 2011 flood, lighter south-easterly winds allowed the flood plume to move eastwards rather than northwards, increasing the length of exposure of sites like Clam Bay and Halfway Island to the floodwaters. The pattern of flood impact clearly depends on the amount and timing of the river discharge and the prevailing wind strength and direction. During summer, large volume flows in the rivers feeding the Fitzroy River caused by monsoonal low pressure systems or cyclones typically bring with them high winds from the south east and heavy seas which, if they coincide with the peak discharge, will naturally limit the amount of impact to the reefs by pushing the plume northwards along the coast.

In the Keppels, the pattern of flood impact appears to explain the geomorphology of the reefs. The buoyant nature of plumes limits the amount of damage to deeper coral communities at sites towards the edge of the plume but severely impacts the expansive shallow reef platforms on southern and western shores of many of the islands. Following flooding, deep reefs that survive the flood event would act as reservoirs of coral larvae that would re-populate denuded reefs during subsequent spawning events. As suggested by Brown et al. [46], shallow platform reefs may be continually in early successional stages with low species diversity but possibly high genetic heterogeneity because they are composed of relatively young populations of principally broadcast spawning corals recruited from multiple adjacent populations. Over time, the mortality caused by the episodic flooding leaves an accumulation of coral rubble on the shores facing the source of the flood plume, forming an ideal substrate for recruitment. After floods these shallow platforms of coral rubble are quickly re-populated by the fast-growing and highly abundant *Acropora* and *Pocillopora* species. Mass mortality from episodic flooding may thus represent a *raison d' être* for the wide, shallow platform reef flats that are typical of the Keppel Bay Islands and that would otherwise be limited by sea level.

The time-frame for recovery of Keppel reefs from the 2011 flood is expected to take ∼10–15 years based on that of the recovery from the 1991 floods. This timeframe is similar to the recovery of mid-shelf GBR reefs from extensive cyclone damage [47] whereas regeneration of coral cover from bleaching in the Keppels in 2006 was ∼1 year [48]. Such dramatic differences in recovery times are influenced by the level of damage to the reef structure, the persistence of coral tissue that survived the disturbance and to the connectivity of impacted reefs to healthy nearby, or deeper, reefs. For instance, another flood or bleaching event in quick succession could delay recovery, allowing macro-algal species to take hold and potentially tipping the balance towards permanent phase shifts [49]. Critically, unlike regeneration after bleaching, when recovery was predominantly from the remaining living coral tissue in a landscape of patchy mortality [48], regeneration from floods relies much more on recruitment when mortality is near total. Until the flood plume dissipates and water quality returns to normal, subsequent spawning events may be affected by poor water quality [50]–[52], some aspects of which can reduce fertilisation [24], [53] and effect recruitment success [54]–[56]. As has occurred in the past, successful and timely regeneration of the Keppel Bay Islands reefs will be dependent on a period of low disturbance, regrowth of coral colonies from surviving tissue and recruitment from adjoining coral communities, particularly those located at connected sites in waters deeper than ∼6 m; suggesting that conservation efforts should be focused on protecting these areas and on improving water quality.

The influence of changing climate patterns on the frequency and size of major floods in the Fitzroy catchment and others like it has implications for the resilience of inshore reefs in the southern GBR. Shallow platform reefs that are typical of the southern and western shores of islands in the Keppels may play an important role in supporting wider resilience because the high light levels common on these reefs supports rapid coral growth and promotes high fecundity, providing an abundant source of new coral recruits once the colonies reach reproductive maturity. In addition, a variety of marine organisms depend on their dense communities of fast-growing and structurally complex acroporid corals for habitat and food [57]. Even the temporary loss of these coral communities would significantly reduce coral recruitment in the years after a flood however there are also likely to be impacts on the fecundity and competitive capacity of surviving coral communities from lingering post-flood hyposaline seawater [58], sediment and nutrients [8]. Fine sediments and nutrients can be easily re-suspended by strong winds and big tides for some time after a major flood until subsequent exchanges with offshore water masses eventually renew the water in the system. It is therefore likely that regeneration will only occur some years after a flood, affecting the resilience of the entire region during that time. A warming climate could mean an increase in the intensity of monsoonal lows [59], decreased annual rainfall and increased summer rainfall intensities [60]. Higher summer rainfall anomalies (50+ mm) are already evident for Australia over the last 100 years [44]. Lower overall annual rainfall followed by intense rain events during summer in GBR catchments such as the Fitzroy may lead to increased sediment runoff due to overall reduced vegetation, potentially increasing the sediment threat to reefs [8], [9]. Imposed over the episodic impacts of flooding is the threat of summer bleaching events [22]. If climate predictions are realised there are likely to be negative consequences on inter-disturbance regeneration of some reefs over time, weakening reef resilience as a whole.

## Conclusions

In Keppel Bay, in the southern GBR, expansive shallow reefs on the leeward sides of the islands are of great importance to tourism because they typically provide easy access for reef-based activities. Because of the episodic nature of cyclonic rain events in the region, the business success of tourism infrastructure established in between flood events can be heavily impacted by the temporary loss of corals after flooding. Meanwhile, the surviving incipient fringing reefs comprised mainly of much slower growing but sediment- and salinity-tolerant non-acroporids become the focus of tourism activities until the platform reefs recover. In order to support tourism in reef regions such as the Keppel Islands it may be necessary to consider human intervention to support the regeneration of the coral cover on high value reefs after major floods. Methods such as *ex-situ* coral culture and transplantation and assisted recruitment [61] could prove critical in the recovery of some reef areas and could support tourism in the short term. However, it must be accepted that such attempts can only be temporary until the next major disturbance. In the long term, it is clear from this study that to support reef resilience, management and conservation efforts should be focused on areas of reef that can survive intermittent natural disturbances rather than only on those that are under pressure from anthropogenic impacts.
